# Dr. Congar’s Private Medical School

**Published:** 1848-01

**Authors:** 


*Dr. Congar's Private Medical School.*—Dr. Congar, whose card will be found upon the advertising pages, proposes to open a private medical school in Buffalo, for the purpose of giving a continued, regular, and thorough course of instruction. Dr. Congar has already had some experience in giving private instruction and examinations, and while he has demonstrated to himself the success of the project, he has also demonstrated to others his complete qualifications for the duty. The facilities which he now offers to his pupils are extraordinary, and ought to ensure him full rooms. The amount which he proposes to charge for a years' tuition—$50, is something more than the student pays whose duty it is to clean office, carry medicines, and collect accounts, but the amount of his acquisitions in medicine and surgery, will, we venture to affirm, be also something greater. *In this way only*, by entering offices where tuition is demanded, and where regular and constant instruction is given, can young men ever be made good students and prepared to practice. The $50 thus paid, is $200 earned, for the student will learn more in one year in such an office, than he *can* learn in three years in the office of any man, however extensive his practice, who leaves his student to instruct himself.

